# Ultra-High Packing Density Next Generation Microtube Array Membrane for Absorption Based Applications

**DOI:** 10.3390/membranes11040273

**Published:** 2021-04-08

**Authors:** Chee Ho Chew, Wan-Ting Huang, Tzu-Sen Yang, Amanda Chen, Yun Ming Wu, Mai-Szu Wu, Chien-Chung Chen

**Affiliations:** 1 Graduate Institute of Biomedical Materials & Tissue Engineering, College of Biomedical Engineering, Taipei Medical University, Taipei 11052, Taiwan; chchew88@gmail.com (C.H.C.); sandyhuang@mtamtech.com (W.-T.H.); nickymwu@ms34.hinet.net (Y.M.W.); 2 Graduate Institute of Biomedical Optomechatronics, Taipei Medical University, Taipei 11052, Taiwan; tsyang@tmu.edu.tw; 3 Department of Biology, University of Washington, Seattle, WA 98195, USA; alc48@uw.edu; 4 Division of Nephrology, Taipei Medical University Shuang Ho Hospital, New Taipei City 23561, Taiwan; maiszuwu@gmail.com; 5 Research Center of Urology and Kidney, Taipei Medical University, Taipei 11052, Taiwan; 6 Masters and Ph.D. Programs of Mind Brain and Consciousness, College of Humanities and Social Sciences, Taipei Medical University, Taipei 11052, Taiwan; 7 Center for Cell Therapy and Regeneration Medicine, Taipei Medical University, Taipei 11052, Taiwan; 8 The Ph.D. Program for Translational Medicine, College of Medical Science and Technology, Taipei Medical University, Taipei 11052, Taiwan; 9 College of Biomedical Engineering, Taipei Medical University, Taipei 11052, Taiwan; 10 College of Medicine, Taipei Medical University, Taipei 11052, Taiwan; 11 College of Pharmacy, Taipei Medical University, Taipei 11052, Taiwan

**Keywords:** ultra-high packing density, microtube array membrane (MTAM), triaxial electrospinning, polymyxin B, sepsis

## Abstract

Previously, we successfully developed an extracorporeal endotoxin removal device (EERD) that is based on the novel next generation alternating microtube array membrane (MTAM-A) that was superior to the commercial equivalent. In this article, we demonstrated multiple different parameter modifications that led to multiple different types of novel new MTAM structures, which ultimately led to the formation of the MTAM-A. Contrary to the single layered MTAM, the MTAM-A series consisted of a superior packing density fiber connected in a double layered, alternating position which allowed for the greater fiber count to be packed per unit area. The respective MTAM variants were electrospun by utilizing our internally developed tri-axial electrospinning set up to produce the novel microstructures as seen in the respective MTAM variants. A key uniqueness of this study is the ability to produce self-arranged fibers into the respective MTAM variants by utilizing a single spinneret, which has not been demonstrated before. Of the MTAM variants, we observed a change in the microstructure from a single layered MTAM to the MTAM-A series when the ratio of surfactant to shell flow rate approaches 1:1.92. MTAM-A registered the greatest surface area of 2.2 times compared to the traditional single layered MTAM, with the greatest tensile strength at 1.02 ± 0.13 MPa and a maximum elongation of 57.70 ± 9.42%. The MTAM-A was selected for downstream immobilization of polymyxin B (PMB) and assembly into our own internally developed and fabricated dialyzer housing. Subsequently, the entire setup was tested with whole blood spiked with endotoxin; and benchmarked against commercial Toraymyxin fibers of the same size. The results demonstrated that the EERD based on the MTAM-A performed superior to that of the commercial equivalent, registering a rapid reduction of 73.18% of endotoxin (vs. Toraymyxin at 38.78%) at time point 15 min and a final total endotoxin removal of 89.43% (vs. Toraymyxin at 65.03%)

## 1. Introduction

Sepsis is a debilitating, life threatening disease that is common within Intensive Care Units (ICU)s. This disease is one of the leading causes of death within ICU, accounting for up to 30% of the total deaths [1,2,3,4]. In the United States alone, this number accounted for 200,000 lives and an economic loss of US$ 20 billion per annum [5]. Etiological speaking, sepsis is a highly complex systemic disease that is caused by the dysfunction and dysregulation of the host immune system towards an infection, which results in multi-organ failure [6,7,8]. Within the highly complex inter-related factors, endotoxin is the principal causative agent in the etiology of sepsis [9,10,11]. Clinically, high levels of endotoxin within the human body are often associated with poor clinical outcome [12,13].

Endotoxins naturally occur within the outer membrane layer of Gram-negative bacteria, and they are released into the surrounding upon the death or bacteria cell lysis [14,15,16]. The majority of sepsis sources within ICUs originate from respiratory tract infections which account for 67.4% of all ICU related sepsis cases, followed by abdomen related infections or translocation of bacteria which accounts for 21.8% of cases [17,18,19]. The presence of endotoxin within the bloodstreams of patients results in symptoms such as fever, nausea, hypotension, shivering, and ultimately shock [14]. With increasing concentrations of endotoxin within the blood of the patients, complications such as endotoxin shock, adult respiratory distress syndrome (ARDS), and disseminated intravascular coagulation (DIC) begin to appear [14,20,21]. In addition, high concentrations of endotoxin are known to be a powerful activator of the kinin system, endothelial cells, leukocytes, and platelets, resulting in the dysregulation of the inflammatory response of the patient and ultimately the above outlined complications [14,22]. Therefore, considering that endotoxin molecules are a powerful trigger for systemic inflammation, which potentially leads to the development of cytokine storm and septic shock [23,24,25]. Consequently, the removal of endotoxin molecules for a patient suffering from sepsis represents one of the latest approaches for the management of sepsis.

Currently, one of the leading commercially available endotoxin removal products is hemoperfusion, an endotoxin removal system known as Toraymyxin. This hemoperfusion system utilizes polymyxin B (PMB), which is an antibiotic with a cationic cyclic polypeptide that has a high selective affinity to endotoxin molecules; that were immobilized via covalent bonding onto the surfaces of polystyrene fibers [26,27]. The PMBs interacted and neutralized the bioactivity of Lipid A that is found on endotoxin molecules by interacting with the amino groups and the phosphate group via ionic and hydrophobic bonds [9,27,28,29]. By effectively binding to endotoxin molecules, it interrupts the biological cascade and effectively arrests the development of sepsis/septic shock.

In Japan, Toraymyxin has been approved for clinical use since 1993 and approved for clinical use in Europe since 1998 [26]. Systemic reviews have suggested that the clinical use of Toraymyxin significantly reduced the mortality of sepsis patients from 61.5% in conventional therapies to 33.5% in Toraymyxin groups [9,30]. In a separate study, the prolonged use of Toraymyxin (median: 5.5 h vs. 2.0 h) significantly reduced the mortality rate of patients from 31.8 to 0.0% (*p* = 0.019) [31]. However, with all the advantages, especially in the significant reduction in mortality associated with the use of Toraymyxin, it is a prohibitively expensive medical device that is not covered by insurers in several countries [1]. Current estimates of the treatment cost for a single sepsis patient are at US$ 20,000 (two units of Toraymyxin/patient), thereby resulting in a large number of patients suffering from sepsis being unable to receive this life-saving therapy [32].

To address this, in our previous work, we developed a new generation of microtube array membrane (MTAM) as a novel new platform solution for the removal of endotoxin [1]. Traditional MTAMs are based on the development carried out in 2012, where we observed, contrary to traditional electrospinning, randomly nonaligned fibers [33,34]. During the development of traditional MTAMs, we observed that when the fibers were collected before the electrospun fibers enter the instability region, and in combination with a rotating drum, it allowed us to produced highly aligned, and one-to-one connected fibers as described previously [35]. Microstructurally, the MTAMs consist of one-to-one connected ultra-thin, individually connected hollow fibers that are arranged in an arrayed formation and with superior surface area for diffusion/absorption [36,37]. The unique microstructure translated to an excellent packing density, short perfusion distance, ease of handling on a macro scale, which allowed this platform technology to be applied in multiple areas ranging from anticancer drug screening [38], endotoxin removal [1], tissue regeneration [39,40,41], microbial fuel cell [42], encapsulated cell therapy [43], and fermentation [44,45,46]. To further improve on the capability of MTAMs, we successfully developed the next generation MTAMs known as MTAM-Alternating (MTAM-A), which has a superior surface area of up to 2.3–2.5 times when compared to traditional MTAMs [1]. Furthermore, the high packing density allowed for significant reduction in the final hemoperfusion device size which could potentially benefit patients, especially in infants and underweight cases through the reduction in extracorporeal blood [1,47,48]. Through the immobilization of PMB onto the surfaces of the MTAM-As (MTAM-A-PMB), we successfully demonstrated the MTAM-A-PMB as a hemoperfusion device for the removal of endotoxins. When compared to Toraymyxin, MTAM-A-PMB hemoperfusion devices demonstrated a significantly faster (15 min) and a higher total endotoxin removal, achieving 89.33% (MTAM-A-PMB) versus 65.52% (Toraymyxin) [1].

In view of the significant advantage conferred by the microstructures of the novel new generation MTAM-A, we strive to demonstrate the precise control and fabrication of several variants of MTAMs (including MTAM-A), which led to the development and application of MTAM-A as a hemoperfusion device.

## 2. Materials and Methods

### 2.1. Tri-Axial Electrospinning of MTAM Variants

**Figure 1:**
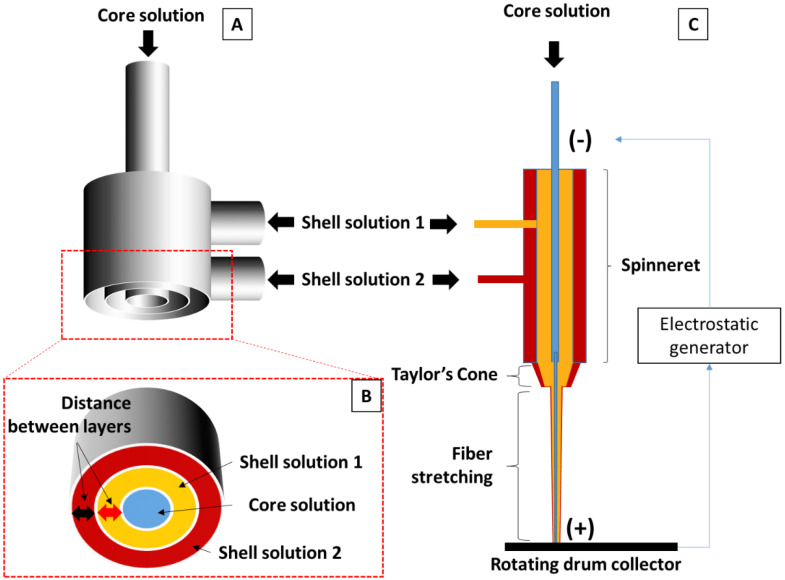
Illustration of the tri-axial electrospinning spinneret (**A**); and the bottom view of the spinneret (**B**). The clearance between layers of 1.0 mm was utilized in this study and indicated in (**B**) as red and black arrows. Schematic illustration of the longitudinal section of the spinneret (**C**).

The solution for the tri-axial electrospinning of the MTAM variants consisted of 3 solutions; namely, a core solution which consisted of 10 wt% polyethylene glycol (PEG, Mw: 35,000; Sigma–Aldrich, Taipei, Taiwan) and polyethylene oxide (PEO, Mw: 900,000; Sigma–Aldrich, Taipei, Taiwan) dissolved in double distilled water (ddH_2_O) until a homogenous solution was obtained. As for the 2 shell solutions, they were similar content wise and were prepared by dissolving 18 wt% polysulfones (PSF, Mw: 35,000; Sigma–Aldrich, Taipei, Taiwan) in a co-solvent of tetrahydrofuran (THF; Sigma–Aldrich, Taipei, Taiwan) and dimethylacetamide (DMAC; Sigma–Aldrich, Taipei, Taiwan) at a solvent ratio of 8:2, under ambient conditions. The resulting solutions were tri-axially electrospun with 3 distinctive spinnerets designed and fabricated internally with varying clearance between the layers of 0.5 mm, 0.8 mm, and 1.0 mm (Figure 1).

The fabrication parameters of the respective MTAM variants were obtained by varying the electrospinning voltage between 4.5–8.5 kV, a spinneret to collector distance of 1.0–3.0 cm, and a collector rotation speed of 60–150 rounds per minute (RPM) under ambient conditions. The resulting fibers (traditional single layered MTAM: PSF MTAM; alternating MTAM: MTAM-A; and double layered MTAM: MTAM-D) were carefully retrieved and transferred into ddH_2_O and soaked for 24 h under ambient conditions. Finally, the respective fibers were air dried for 48 h under ambient conditions and stored at 4 degrees Celsius.

In the case of hairy MTAM (MTAM-H), the introduction of nano-scaled hairy structures into the respective lumens of the MTAM-H was achieved by modifying the core solution. Instead of the core solution outlined above, a modified core solution which consisted of 6 wt% PSF (Mw: 35,000; Sigma–Aldrich, Taipei, Taiwan), a surfactant (PEG 40; Sigma–Aldrich, Taipei, Taiwan); and the core solution outlined at a ratio of 1:5:10 that was sonicated for 2 h and electrospun immediately. The remainder of the solution and fabrication parameters were similar to those outlined above.

### 2.2. Microstructure Analysis of the Respective MTAM Variants

The respective variants of MTAMs were soaked in liquid nitrogen for 300 s and freeze-fractured with the sharp edge of a razor. Next, the respective membranes were sputter coated with 99.0% gold for 160 s. The resulting sputter coated membranes were characterized via scanning electron microscopy (SEM; S-2400 Hitachi, Tokyo, Japan) at 15 kV, and microstructures were analyzed via Image J analytical software (NIH, Bethesda, MD, USA). To determine the surface area of the respective MTAM variants, the outline of the inner lumen was selected and quantified. The resulting area was then multiplied by the number of lumen counts per 200 µm, and the resulting area per 200 µm was determined and cross compared against the respective variants of MTAMs.

### 2.3. Contact Angle Determination of Double Distilled Water (ddH_2_O) on the Surfaces of the Respective MTAM Variants

The MTAM variants were cut into dimensions of 1 cm × 3 cm. Next, the cut membranes were carefully adhered to standard microscope slides (Biomann Scientific, Taipei, Taiwan) with double sided tape. The entire setup was placed onto the stage of the angle goniometer (Digidrop PROD, GBX, Paris, France) and secured in place. Next, 5 µL of ddH_2_O was carefully extruded from the in-built glass syringe to form a droplet on the tip of the syringe needle, and the automated measuring system was engaged, resulting in the transfer of the ddH_2_O droplet from the syringe needle tip onto the surface of the respective membranes. The image of the resulting water droplet on the surface of the membranes was captured via the in-built camera, and the contact angle measured and analyzed via Image J analytical software (NIH, MD, USA)

### 2.4. Mechanical Properties of the MTAM Variants

Samples of the respective membranes were cut into rectangular dimensions measuring 1 cm × 6 cm. The short edges of the membranes were securely clamped in the holder of the universal testing machine (LF Plus Testing Machine; LLYOD Company, West Sussex, UK), and the automated testing was engaged under constant load. Based on the obtained data, a stress–strain (SS) curve was plotted for each of the respective membranes, and the maximum tensile strength and Young’s modulus were determined accordingly.

### 2.5. Immobilization of the Polymyxin B (PMB) onto the Surfaces of PSF MTAM-A and the Determination of Degree of Immobilization Rate of PMB

The immobilization process was conducted as previously described [1,49,50]. Precut PSF MTAMs were treated with acetic acid (AA; Sigma–Aldrich, Taipei, Taiwan) plasma at 100 millitorr (mTorr), at a power of 10 watts (W) for 5 min. Next, the AA treated PSF MTAM-A were soaked in 10 wt% acrylic and ammonium persulfate (APS; Sigma–Aldrich, St. Louis, MO, USA) solution and subsequently treated with 4 mL of Vitamin B2 (antioxidant; Sigma–Aldrich, St. Louis, MO, USA). The treated PSF MTAM-As were then transferred into internally developed grafting equipment developed by Professor Ko-Shao Chen of the Institute of Materials Engineering, Tatung University, Taiwan, which was followed by a system purge with nitrogen gas for 10 min (Shen-Yi, Taipei, Taiwan). Next, 12 min of UV irradiation (365 nm) at 2000 W was administered; and upon completion of irradiation, the respective PSF MTAM-As were transferred into ddH_2_O for a 10-min soak. After which, the PSF MTAM-As were soaked in 1-Ethyl-3-(3-dimethylaminopropyl) carbodiimide (EDC; Sigma–Aldrich, St. Louis, MO, USA)/N-Hydroxysuccinimide (NHS; Sigma–Aldrich, St. Louis, MO, USA) solution (pH: 5.5–5.9) for 2 h; and finally, in a PMB solution for 10 min. The resulting crosslinked membranes were air-dried under ambient conditions and stored at 4 degrees Celsius for downstream use.

To determine the degree of immobilization of PMB, pre- and post-treated PSF MTAMs were freeze-dried for 24 h (Kingmech, Taipei, Taiwan). The freeze-dried membranes were weighed, and the degree of immobilization of PMBs was determined as follows:

$$I=\left[\frac{Wb-Wa}{Wa}\right]\times 100\%$$

where, 

*I* = Immobilization rate of polymyxin B (PMB)

*Wb* = Weight of PSF MTAM-A-PMB membranes after crosslinking

*Wa* = Weight of PSF MTAM-A membrane before crosslinking

Next, Fourier transform infrared (FTIR; ThermoFisher, Waltham, MA, USA) tests were carried out to confirm the successful crosslinking of PMB onto PSF MTAM-As, with the transmission spectra scanned from the wavelength 400 cm^−1^–4000 cm^−1^

### 2.6. Design, Fabrication, and Assembly of the PSF MTAM-A-PMB Based Hemoperfusion Unit

Internally designed housing of the hemoperfusion device was carried out with Inventor 2019 (Autodesk, Mill Valley, CA, USA). Briefly, the designed housing consisted of 2 inlets for the entry and exit of blood, with the center wall intentionally removed to provide a window view measuring 1 cm × 1.5 cm of the PSF MTAM-A-PMB when the dynamic absorption test was carried out. The resulting design was 3D printed (Form 2, Formlab, Taipei, Taiwan), followed by a downstream UV curing treatment for 15 min and a 15 minutes’ soak in -sopropyl alcohol (IPA, Sigma–Aldrich, St. Louis, MO, USA) to remove excessive resin residues. 

Next, varying quantities of PSF MTAM-A-PMBs were precut into a dimension of 1 cm × 8 cm and staked into a bundle. Epoxy putty (Slink, ECP-1305, Boston, MA, USA) was carefully spread at the edges, between the layers of the bundle of PSF-MTAM-PMB. Finally, the entire membrane bundle was introduced into the above designed and fabricated housing, and with the aid of an internally designed centrifugal device, potted with additional epoxy putty. The cured ends of the hemoperfusion device were then cleanly cut off with a fresh blade to ensure a smooth and consistent opening at the ends of the membrane bundle. Finally, the respective end caps of the hemoperfusion unit were attached.

### 2.7. Dynamic Adsorption Testing of the Removal of Endotoxin with the PSF MTAM-A-PMB Hemoperfusion Device

To dynamically test the performance of the PSF MTAM-A-PMB hemoperfusion unit, a circuit was prepared. Briefly, the hemoperfusion unit was attached via standard silicon tubing (Biomann Scientific, Taipei, Taiwan) to a rotary pump (Biomann Scientific, Taipei, Taiwan) and blood reservoir to mimic the body of the patient, forming a closed loop. Next, the entire system was primed with a sterile saline solution that was spiked with heparin (10 wt%, Sigma–Aldrich, St. Louis, MO, USA) and completely drained. Whole blood containing 100 EU/mL of endotoxin (Sigma–Aldrich, St. Louis, MO, USA) was transferred to the blood reservoir in the above circuit. The rotary pump was turned on at set as a flow rate of 2 mL/minute, and whole blood samples were collected at the predetermined time points for limulus amebocyte lysate (LAL) and hematological studies.

To quantify the quantity of endotoxin in any given sample, the ETOXATE kit (Sigma–Aldrich, St. Louis, MO, USA) was utilized. One hundred microliters of plasma for the collected samples was transferred into a depyrogenated test tube that was validated to be free from endotoxin (Techno Plastic Products, Trasadingen, Switzerland) that contained an equal volume of working reagent. Next, the respective test tubes were incubated at 37 degrees Celsius in an undisturbed water bath (Biomann Scientific, Taipei, Taiwan) and allowed to sit undisturbed for 60 min. Next, the respective test tubes were gently inverted and left to stand on their caps. Positive gelation was indicated by the ability of the gel to adhere to the top of the inverted test tube, indicating the presence of endotoxins. 

For hematological studies, 3 mL of whole blood samples were collected at the predetermined time points and immediately delivered to Union Clinical Laboratory (UCL; Taipei, Taiwan) and/or the Pathology Department of Taipei Medical University Hospital (Taipei, Taiwan) for analysis.

## 3. Results

**Figure 2:**
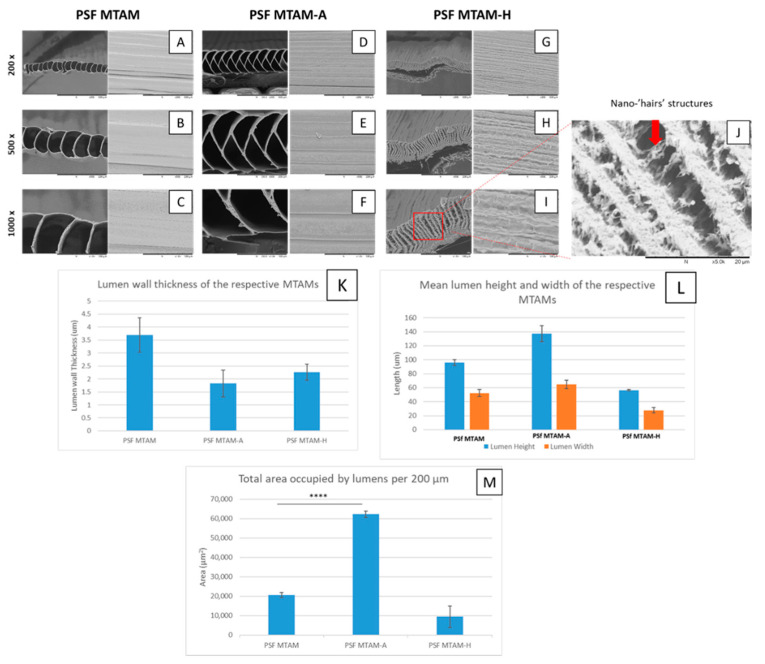
(**A**–**C**) Scanning electron micrograph of the traditional single layered polysulfone microtube array membranes (PSF MTAMs); (**D**–**F**) the novel new alternating layered polysulfone alternating microtube array membranes (PSF MTAM-A); and the single layered PSF MTAM with nano-hair structure introduced into the respective lumens (**G**–**I**). The respective microstructures of the MTAM variants were presented in (**J**) lumen wall thickness; and (**K**) the mean lumen dimension. (**L**). The total area occupied by lumens (**M**) The novel new PSF MTAM-A was found to have a significantly higher surface area when compared to the traditional PSF MTAMs. For the downstream toxin removal from whole blood applications, the polysulfone hairy microtube array membranes (PSF MTAM-H) will not be considered as the lumen dimension was simply too narrow for red blood cells to pass through unharmed. Parts of the figure reproduced with permission from John Wiley and Sons. (Scale Bar: Yellow 500 µm, Red 200 µm, and Orange 100 µm). Statistical definition: *p*-value ≤ 0.001 (****).

Three distinctive variants of MTAMs were successfully obtained as seen in Figure 2; namely, the traditional single layered PSF MTAM (Figure 2A–C); alternating layered MTAM (PSF MTAM-A) with trapezium shaped lumens arranged in an alternating arrayed formation (Figure 2D–F); and the single layered MTAM with hairy nanostructures introduced (Figure 2G–K). Of the respective MTAM variants, the PSF MTAM-H registered the lowest total area occupied by lumens per 200 µm of 9408.56 ± 544.34 µm^2^. On the contrary, the MTAM variant with the greatest total volume occupied by lumen per 200 µm was PSF MTAM-A, which registered a value of 62,254.51 ± 1636.62 µm^2^ which was statistically greater than those of the traditional single layered PSF MTAM at 20,644.13 ± 1325.39 µm^2^.

**Figure 3:**
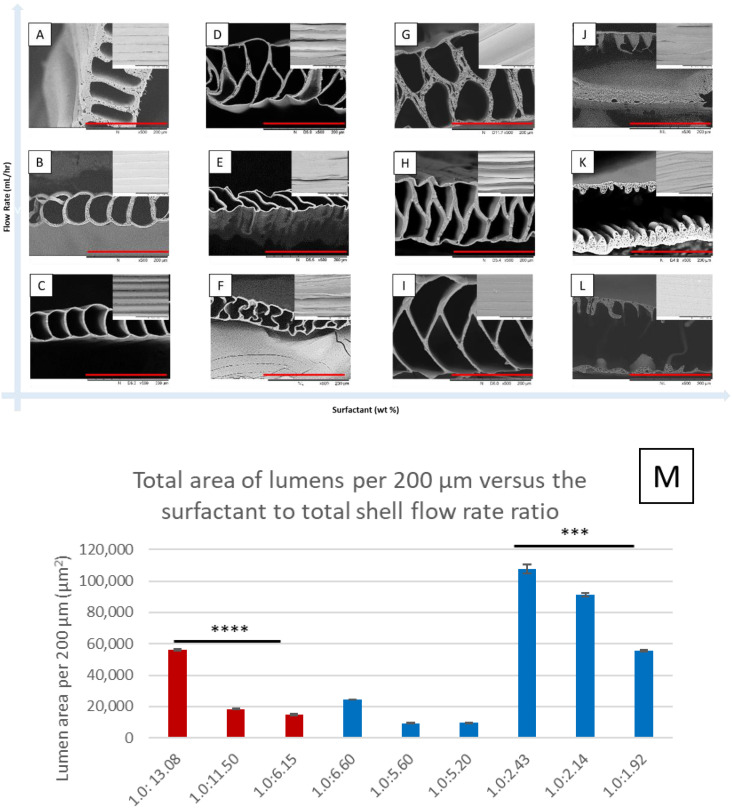
Scanning electron microscopy (SEM) of the respective membranes obtained through the modification of the total shell flow rate versus the weight percentage of surfactant. Respective parameters are found in Table 1. All membranes were tri-axially electrospun under ambient conditions with a spinneret to distance collector of 3 cm and collected by a rotating drum set at 90 ± 10 rpm. Generally speaking, at a low surfactant concentration and an increasing total shell flow rate, the lumen wall thickness increased (**A**–**C**); when the surfactant concentration increased, a set of membranes with ‘soft’ and unstable microstructures was observed (**D**–**F**). Above a certain threshold of surfactant concentration, the alternating PSF MTAM-A was obtained with the respective thickness of the lumen wall registering a higher reading as the total shell flow rate was increased (**G**–**I**). Ultimately, when the surfactant concentration was increased beyond the stable PSF MTAM-A forming formulation, membranes without vertical walls were observed along with a center mass, which increased with increasing flow rate (**J**–**L**). The total surface area of lumens per 200 µm and the corresponding surfactant to total shell flow rate as seen in Table 1 (**M**). Based on the two distinct membrane groups, namely **A**–**C** and **G**–**I**, internally within the respective groups, the reducing surfactant to total shell flow rate ratio translated to a reduction in the total surface area occupied by the respective lumens. Red colored bars are the membranes produced with the core flow rate of 7.0 mL/h, while blue colored bars are the membranes produced with the core flow rate of 8.0 mL/hr. Parts of the figure reproduced with permission from John Wiley and Sons. (Scale bar in red: 200 µm). Statistical definition: *p*-value ≤ 0.05 (***); *p*-value ≤ 0.001 (****).

Next, we discovered that the formation of the novel new microstructure appeared to be directly tied to the percentage of surfactant utilized in the solution parameter. At low concentrations of < 2.5 wt%, the single layered MTAM variant was obtained, although with some degree of variation in terms of the integrity of the individual lumen, which fared better at lower surfactant wt% as seen in Figure 3A–C as opposed to those seen in Figure 3D–F. Interestingly, at the surfactant concentration of 2.5 wt% and a flow rate of 16.5 mL/h, the pseudo-alternating MTAM was obtained, which possessed regions of improperly formed alternating microstructures (Figure 3D). True PSF MTAM-As were obtained when the surfactant concentration of >7.0 wt% was utilized, in combination with the high shell flow rate (Figure 3G–I). If the concentration of surfactant (<8.0 wt%) and in combination with a high total shell flow rate of >17 mL/h was utilized, a new variant of MTAM without vertical walls and potentially a solid center mass was obtained (Figure 3J–K). Interestingly, the size of this solid center mass appeared to be proportional to the flow rate, which was extremely evident in the case of Figure 3J. 

Based on the outcome in the difference in microstructures for the respective variants of MTAMs as seen in Figure 3, we discovered a trend in which the ratio of the surfactant concentration to the total shell flow rate appeared to change from single layered MTAMs to alternating layered MTAMs when the ratio decreased (Table 1). Hence, we also analyzed the effect of the ratio of the surfactant weight percentage to the total shell flow rate on the surface area of the respective variants of MTAMs, and we discovered that for the respective MTAM variants, the surface area appeared to decrease (Figure 3M and Table 1).

**Figure 4:**
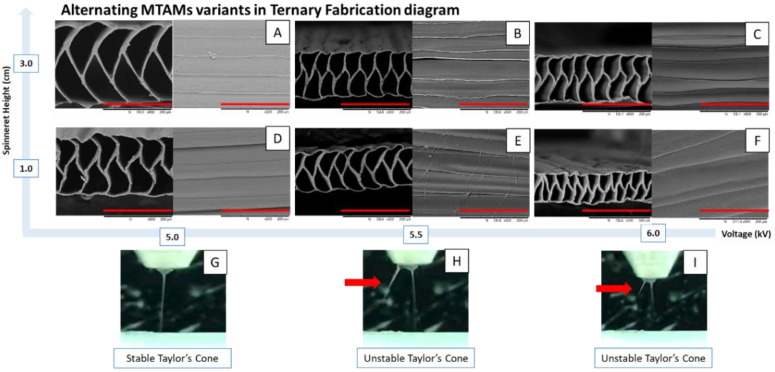
Effects of the voltage and the corresponding Taylor’s cone. (**A**) the optimum PSF MTAM-A with trapezium shaped lumens arranged in an arrayed formation. (**B**–**F**) PSF MTAM-A that are beyond the stable range of the formation of PSF MTAM. With increasing voltage, the ‘flight time’ of polymers during electrospinning are significantly reduced and resulting in less time for solvent to evaporate and thereby resulting in wavy vertical lumen walls. In addition, beyond the stable formation of PSF MTAM-A (**A**), the top view of the respective fibers began to lose its consistency in terms of fiber diameter, which was extremely evident in (**B**,**C**,**E**,**F**). (**G**–**I**) Through the increase in voltage, the formation of rouge fibers was observed (red arrows), which separated from the main Taylor’s cone, potentially resulting in fibers seen in the top view of the SEMs (**E**,**F**). Parts of the figure reproduced with permission from John Wiley and Sons. (Scale bar in red: 200 µm).

Within the parameters for the fabrication of the PSF MTAM-As, several variants of it were obtained through the manipulation of spinneret height to collector and voltage (Figure 4). Based on these data, it was observed that the PSF MTAM-A that was fabricated at a spinneret height of 3.0 cm and a voltage of 5.0 kV under ambient conditions produced the best membranes with excellent evenness and consistent trapezium shaped fibers (Figure 4A). At a lower spinneret height, the respective lumens of the membrane lost the trapezium shape and were replaced with wavy, vertical walls (Figure 4D). At higher voltages, the consistency of the lumen width began to vary, as seen in the top view of the respective SEMs (Figure 4B,C,E,F). Furthermore, fine random fibers existed for the membrane fabrication at a spinneret height of 1.0 cm and a voltage of 5.5 kV (Figure 4E). Rouge side arching of fibers was observed at Taylor’s cone, as seen in Figure 4H,I.

**Figure 5:**
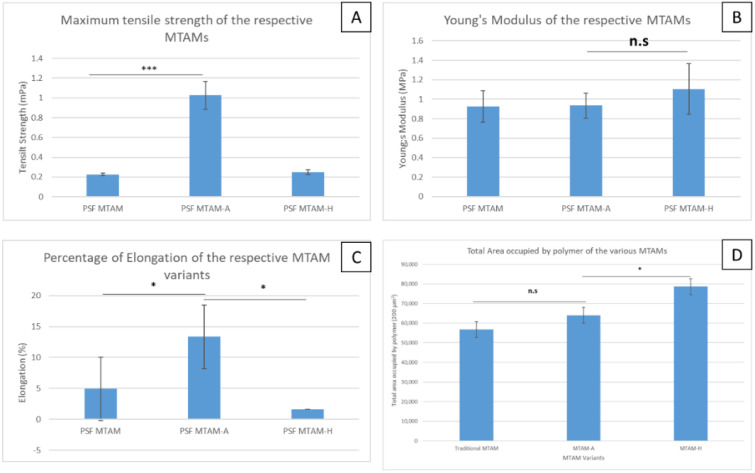
Mechanical properties of the respective MTAM variants (Maximum tensile strength, A; Young’s modulus, B; Percentage of elongation, C; and total area occupied by polymer per 200 µm). PSF MTAM-A registered the highest maximum tensile strength (1.02 ± 0.13 MPa) that was superior to PSF MTAM (**A**). No significant differences in terms of Young’s modulus were observed between the respective MTAM variants (**B**); while the PSF MTAM-A registered a greater degree of elongation before failure when compared to the respective MTAM variants (**C**). Interestingly, the total area occupied by polymer per transverse section that was subjected to stress during the mechanical testing registered no significant difference between the traditional PSF MTAM and PSF MTAM-A (**D**); while the PSF MTAM-H registered the greatest value in part due to the narrow lumens which were not beneficial for our downstream applications (**D**). Statistical definition: not significant (n.s); *p*-value ≤ 0.5 (*); *p*-value ≤ 0.05 (***).

Mechanically, the PSF MTAM-A registered the greatest degree of tensile strength at a value of 1.02 ± 0.13 MPa (Figure 5A). This was statistically greater than those of the PSF MTAM at 0.22 ± 0.01 MPa. In terms of Young’s modulus, no significant differences were observed between the respective variants of the MTAMs, with the PSF MTAM registering a value at 0.92 ± 0.16 MPa, and PSF MTAM-A at 0.93 ± 0.12 MPa. Interestingly, the PSF MTAM-A also registered the greatest degree of elongation of up to 57.70 ± 9.42% (Figure 5C) despite having the total area occupied by polymer, which was load bearing somewhat similar to those of PSF MTAM and PSF MTAM-A (Figure 5D) and equal Young’s moduli (Figure 5B).

**Figure 6:**
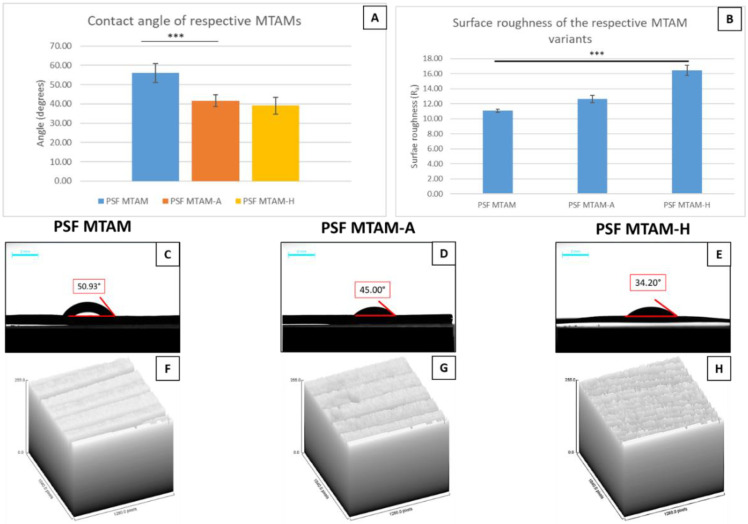
Contact angle of double distilled water on the respective MTAM variants (**A**); and the surface roughness of the surfaces of MTAM variants (**B**). Macroscopic images of the respective double distilled water droplet on the surfaces of the MTAM variants (**C**–**E**); and the corresponding surface plot generated by the Image J analytical software (**F**–**H**). The PSF MTAM-H was observed to have the surface with the greatest roughness (**E**,**H**), and as well as a significantly higher frequency of ‘peaks’ and ‘valleys’ (**H**), resulting in the lowest contact angle among the respective MTAM variants. Statistical definition: *p*-value ≤ 0.05 (***).

In terms of the double-distilled water contact angle, there was a significant difference in terms of the value of PSF MTAM and PSF MTAM-A (61.0 ± 3.4° vs. 41.6 ± 3.1°) (Figure 6A). Comparatively, the surface roughness (R_a_) of the PSF MTAM-H registered the highest value at 16.53 ± 0.68 µm, as opposed to PSF MTAM-A, which registered a value of 12.63 ± 0.67 µm (Figure 6B). These readings were statistically significant when compared to those of PSF MTAM at 11.07 ± 0.21 µm, respectively. Additionally, this finding appeared to correlate to the top view of the SEM of the respective MTAM variants seen in Figure 2A–I, which revealed excellent consistency of fibers for both PSF MTAM and PSF MTAM-A; while PSF MTAM-H revealed highly narrowed, inconsistent, and rough surfaces (Figure 2G–H)

**Figure 7:**
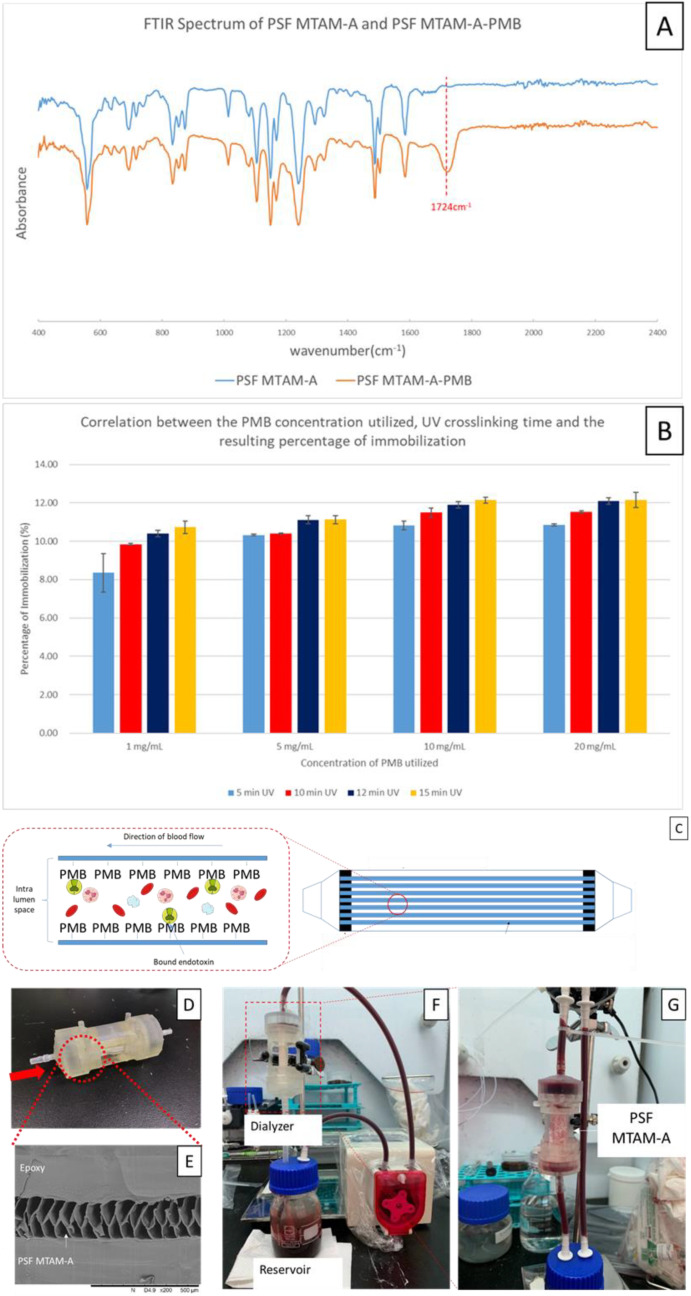
Fourier transform infrared spectroscopy (FTIR) of the PSF MTAM-A and PSF MTAM-A-PMB with a distinctive peak at the amidoxime bond between PMB and 1-ethyl-3-(3-dimethylaminopropyl) carbodiimide (EDC) clearly observed at spectra wavelength 1724 cm^−1^ (**A**). Immobilization efficiency of PMB at various concentrations versus UV exposure time (**B**). The highest immobilization efficiency of PMB was found to be at 10 mg/mL at 12 min of UV exposure, achieving 11.90%. Schematic illustration of the PSF MTAM-A-PMB (**C**). Image of the assembled PSF MTAM-A-PMB based hemoperfusion unit (**D**) and the corresponding SEM of the potting end indicated no microscopic pores, which might potentially serve as leakage sites (**E**). Image of the actual dynamic dialysis process with whole blood spiked with 100 EU endotoxin (**F**,**G**). Parts of the figures reproduced with permission from John Wiley and Sons.

As the PSF MTAM-A registered the greatest surface area (Figure 2N) and excellent microstructural properties, PSF MTAM-As were selected for downstream immobilization with PMB and testing for the ability/performance of endotoxin removal. Fourier transform infrared spectroscopy (FTIR) analysis of both the PSF MTAM-A and PSF MTAM-A-PMB revealed a distinct peak at 1724 cm^−1^, which was known to be the amidoxime bond (-CONH) of EDC and polymyxin B (PMB). As the key endotoxin absorbing molecule, PMB is relatively costly. It is of utmost importance that the minimal amount of it is used while maximizing the performance of the endotoxin removal capacity. We determined that the highest immobilization efficiency was at 10 mg/mL of PMB with the crosslinking UV exposure duration of 12 min (Figure 7B). The resulting membranes (PSF MTAM-A-PMB) were assembled via epoxy potting into our own internally designed and 3D printed (Figure 7D) housing (hereinafter known as ‘hemoperfusion unit’). SEM examination of the potted ends of the hemoperfusion unit, as seen in Figure 7E, were defect free, which ensured that all blood flowed into the respective lumens during testing, as seen in Figure 7G. A further confirmation that the blood only flowed inside the lumens of the PSF MTAM-A-PMBs was obtained when the dynamic absorption of endotoxins via the hemoperfusion unit was carried out, which revealed no blood on the outside of the fibers in the center regions of the hemoperfusion units (center housing wall intentionally removed for easy observation; Figure 7G). 

**Figure 8:**
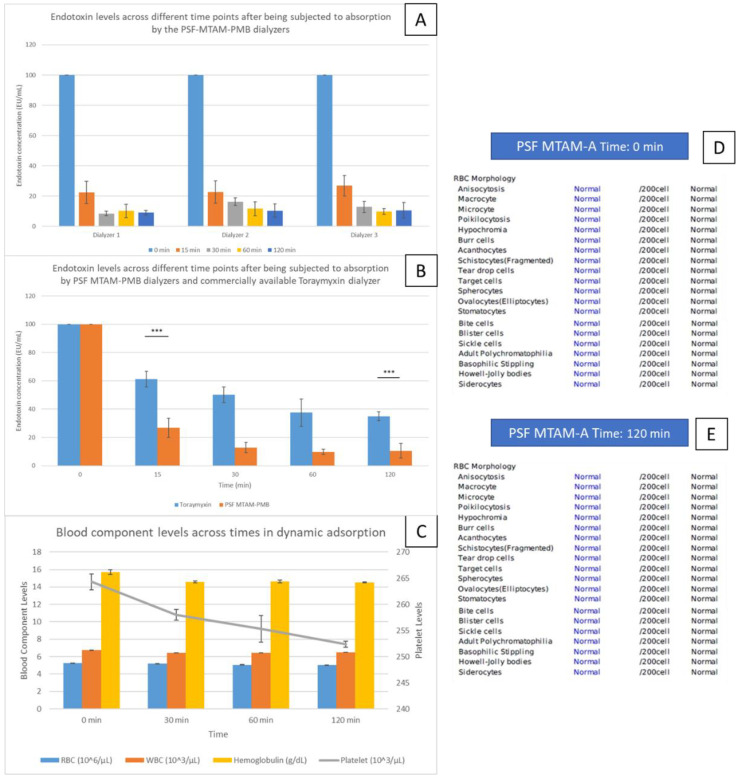
Endotoxin levels across different time points of three samples of the PSF MTAM-A-PMB based hemoperfusion unit (**A**); n = 3 for each sample point). Comparison between the performance of the commercially available Toraymyxin endotoxin removal system versus the PSF MTAM-A-PMB based hemoperfusion unit (internally conducted). (**B**) The performance of the PSF MTAM-A-PMB hemoperfusion system achieved a rapid removal of up to 73.18%, while the commercial latter registered a value of 38.78%. The total removal capacity of the PSF MTAM-A-PMB hemoperfusion system was 89.43%, compared to the commercial latter, which was 65.03%. Changes in blood components across time (**C**). Pathological analysis conducted at Union Clinical Laboratory (UCL) of the red blood cell morphology per and post absorption (**D**,**E**). Statistical definition: *p*-value ≤ 0.05 (***).

To demonstrate the reproducibility of the capability of the respective hemoperfusion unit in the absorption removal of endotoxin, three hemoperfusion units were subjected to dynamic testing with whole blood spiked with endotoxin. Consistent across all three units, a significant drop in endotoxin levels was observed at time point 15 min, which registered a significant drop of 77.70% to 22.31 ± 7.38 EU/mL, and this number continued to drop to 8.95 ± 1.53 EU/mL at time point 120 min (Figure 8A). This trend was similar when a comparison between endotoxin performance removal between the commercially available Toraymyxin and our PSF MTAM-A-PMB hemoperfusion unit was made (Figure 8B). At 15 min, the endotoxin removal of Toraymyxin was 38.78% as opposed to the PSF MTAM-A-PMB based hemoperfusion unit, which registered a rapid reduction in endotoxin level of 73.18%. Ultimately, the system developed in this study registered a total endotoxin removal capacity of 89.43%, as opposed to those of Toraymyxin, which registered a reduction of 65.03%. 

Hematologically speaking, the reduction in red blood cells (RBC), white blood cells (WBC), platelet, and hemoglobulin registered a reduction of 4.42% from 5.23 ± 0.02 × 10^6^/µL to 5.01 ± 0.01 × 10^6^/µL; 3.86% from 6.73 ± 0.02 × 10^3^/µL to 6.47 ± 0.01 × 10^3^/µL; 4.53% from 264.33 ± 1.52 × 10^3^/µL to 252.33 ± 0.57 × 10^3^/µL; and 7.4% from 15.70 ± 0.26 g/dL to 14.63 ± 0.15 g/dL (Figure 8C); which were well within the safety limits. Analysis of the RBC morphology by the Union Clinical Laboratory found no abnormalities. 

## 4. Discussion

In this work, the primary goal was to develop a new generation of MTAM variants with superior surface area for immobilization of absorbing molecules for the removal of endotoxin, which served as a model. Unlike our previous work and also other core shell electrospinning, which utilized the coaxial electrospinning system [51], we utilized the tri-axial electrospinning spinneret (Figure 1) as the high flow rate required to fabricate these novel new microstructures is simply limited by the clearance of the spinneret, which became a limiting factor at higher flow rates thereby limiting the flow rate at the spinneret tip [52]. 

Based on tri-axial electrospinning, we successfully obtained three variants of MTAMs: the traditional single layered PSF MTAM, which was similar to those reported in our previous works [36,38,42]; the novel new alternating PSF MTAM-A; and the new PSF MTAM-H (Figure 2). Among these MTAM variants, the novel new PSF MTAM-A registered the greatest total area occupied by lumen per 200 µm, which was extremely beneficial in this series of work (Figure 2N). A direct comparison between PSF MTAM-A and PSF MTAM revealed a superior surface area of up to 2.2×, which was consistent with our previous work [1]. By maximizing the amount of surface area that is functional for downstream applications, the PSF MTAM-A provided an unprecedentedly high amount of packing density, which is beneficial in medical applications, especially in this series of work of endotoxin removal where the minimization of extracorporeal blood is beneficial for patients, minimizing the risk of shock or sudden rapid changes to blood parameters [53]. On the other hand, the PSF MTAM-H is uniquely novel but unsuitable in the downstream goal of this series of studies where the PSF MTAM-H was unsuitable for downstream dynamic absorption applications as it potentially results in greater sheer force which will cause RBC rupture (Figure 4J–L) [54,55,56]. This, in turn, will result in the triggering of the coagulation cascade procoagulant state, and hence PSF MTAM-H was excluded from downstream applications [57,58].

Based on the solution parameters in Table 1, we observed a trend in which as the ratio of surfactant weight percentage to the total shell flow rate reduced, the resulting microstructure changed from the traditional single layered PSF MTAM (Figure 3A–C) to the novel new PSF MTAM-A (Figure 3G–I) and beyond. When the concentration of the surfactant approached 7.0 wt%, the novel new PSF MTAM-A was obtained, and we postulated that this was the result of the formation of critical micelle concentration (CMC), which ultimately formed tubular micelles that were arranged in alternating configurations as seen in previous works [59,60]. When the concentration of surfactant was further increased, and in combination with an even higher total shell flow rate, a huge center mass resulted, as seen in Figure 3J–L. 

**Figure 9:**
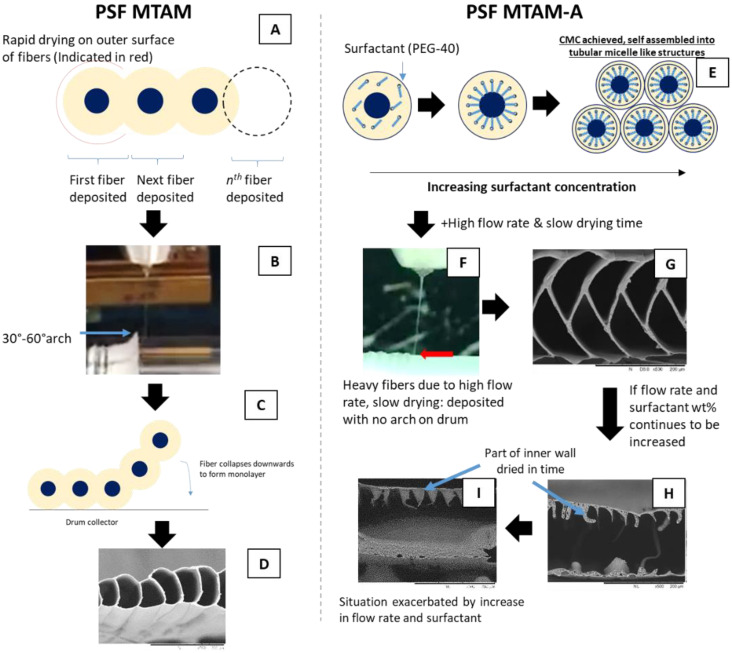
Illustration of the potential formation mechanisms of the respective MTAM variants. Traditional single layered PSF MTAM (**A–D**). PSF MTAM-As were primarily formed through the combination of a high flow rate, in conjunction with a high wt% of surfactants, in which the critical micelle concentration was achieved, resulting in rearrangement and self-assembled into tubule-like micelle structures microemulsion (**E–G**). If the concentration of the surfactant and flow rate was further increased, the vertical walls collapsed, and formed into a large single mass in the center **(H**,**I**).

The size of this center mass was proportional to the total shell flow rate and in combination with the increasing percentage of surfactant [51,53]. In addition, from the respective SEMs, we observed that at higher shell flow rates, the resulting lumen wall thickness of the respective MTAMs was thicker, and this coincided with previous works, which correlated the increase in fiber lumen thickness to the flow rate utilized [61,62]. An illustration of the postulated formation of the respective MTAM variants can be seen in Figure 9. Furthermore, we observed that within the respective sets of MTAMs, which were fabricated using the same core flow rate (Table 1), the relationship of the surfactant weight percentage to the total shell flow rate ratio was proportional to the resulting lumen surface area per 200 µm; within the respective membrane series, the lower the ratio of the surfactant weight percentage to total shell flow ratio resulted in lower functional surface area (Figure 3M).

As seen in Figure 4, several variants of PSF MTAM-A with microstructural differences that corresponded to the changes in the spinneret height and voltage were observed. The optimum shaped PSF MTAM-As were those of trapezium shaped lumens (Figure 4A) that were produced at a voltage of 5.0 kV and a spinneret height of 3 cm. At a lower spinneret height, the vertical walls changed to a wavy-like structure (Figure 4D), and this was mainly due to the reduced spinneret height, which increased the electrostatic force on the polymer, thereby reducing the ‘time in flight’. Consequently, there was a significant reduction in drying duration, thereby impacting the drum collector while the polymer was still relatively soft [63,64]. By increasing the voltage, the individual fibers began to lose their consistency (Figure 4B,C), and when the spinneret height was reduced, the compounded effects resulted in electrostatic potential on the polymers at Taylor’s cone were higher than those required for stable electrospinning; as seen in the formation of rouge fibers (Figure 4H,I) which resulted in random nanofiber on the top of the PSF MTAM-A (Figure 4E). This observation can be explained by the combination of the above electrostatic potential that was above the stable fabrication parameter, which resulted in a rouge electric field; and also the ultra-fast formation of axis oscillation of Coulombic force at different regions of the spinning solution in flight which exerted inertial effect which resulted in the loss of consistency in individual fibers [65,66,67,68,69].

Mechanically, the PSF MTAM-A registered the highest maximum tensile strength (Figure 5A). We postulate that the excellent tensile strength was the result of the alternating formation which redirected that stress/load on the membrane along the diagonally shaped lumen, thereby dividing the force along two intersecting, overlapping lumens, as opposed to those which have either vertical wall (PSF MTAM] [70]. In terms of elongation, the PSF MTAM-A registered the greatest degree of elongation (Figure 5C). Interestingly, the corresponding area occupied by the polymer that was load bearing revealed no significant difference, and Young’s modulus between the PSF MTAM-A and the other MTAM variants revealed no significant differences (Figure 5B,C). This led us to believe the excellent elongation and tensile strength of the PSF MTAM-A was the result of the unique intersecting microstructure which distributed the load, as described earlier, and the result of good crystallization due to stable electrospinning parameters and sufficient evaporation time (outlined above) [71,72,73].

In Figure 6, it was observed that the greatest degree of water contact angle was in the PSF MTAM variant at 61.1 ± 3.5°, and this value coincided with the value found in work by other groups [74]. On the contrary, PSF MTAM-A and PSF MTAM-H registered a significantly lower water contact angle despite being made of the same material. Such findings could be because of the significantly rougher surface of PSF MTAM-A and PSF MTAM-H (Figure 6B), which might be the result of the rounder lumens on the outer surfaces (Figure 3I) and in the case of PSF MTAM-H, a very rough and uneven surface which will cause the water droplet to come into contact with the surrounding ‘protruding’ microstructure, and the surface tension will pull the water droplet outwards, thereby resulting in the reduced water contact angle [75,76,77].

As the PSF MTAM-A registered the greatest surface area, it was selected for downstream immobilization of PMB and endotoxin absorption testing. The FTIR analysis of the PSF MTAM-A-PMB revealed a distinctive peak at 1724 cm^−1^, which coincided with the amidoxime bond of EDC molecule the PMB molecule on the surfaces (Figure 7A) [78]. The crosslinking of PMB resulted in the reduction in the water contact angle as reported in previous work [1], which improves the wettability of polysulfone through the presence of hydroxyl chains on the PMB molecule. Ultimately, this will allow for better contact of the whole blood with the endotoxin absorbing molecules in the downstream application. In the degree of immobilization, it was of utmost importance that parameters be optimized considering that the PMBs are extremely expensive, which will directly impact any future medical devices developed based on this study. To optimize the parameters, we identified that the PMB at a concentration of 10 mg/mL (Figure 7B) with a UV exposure duration of 12 min registered the greatest degree of immobilization. It should be noted that the shortest possible UV exposure must be utilized considering that the 2000 W, high power UV was observed to be capable of damaging the PSF MTAM-A with long term exposure (data not shown). 

We designed the housing of the 3D hemoperfusion unit with the walls intentionally removed to allow us to observe for any extra-luminal blood leakage (Figure 7F). To ensure that there were no leaks between the bundles of PSF MTAM-A-PMBs, special care was paid in applying a generous amount of epoxy to these regions. The entire membrane bundles potted and cut in a swift stroke of the blade, and this retained the openings and integrity of ends of the potted PSF MTAM-A-PMB (Figure 7E). The lack of microscopic defects as seen here was of utmost importance to ensure whole blood only traveled through the luminal space of the respective PSF MTAM-A-PMBs. Compared to a conventional hemoperfusion unit, the PSF MTAM-A-PMB system was intentionally designed to be small so as to minimize the extracorporeal blood of future patients, which will reduce incidences of thermal imbalances and hypotension [79,80]. In the actual dynamic endotoxin absorption process, it was critical that the entire system be primed with sterile saline spiked with 10% heparin as this reduced the amount of blood component loss in the wetting of the material and well as incidences of hemolysis [81,82] and clot buildups that were the result of intraluminal sheer stress [83,84]. At time point 120 min, the absence of blood pooling in the extraluminal chamber of the hemoperfusion system suggested that the PSF MTAM-A-PMBs remained intact, and the potted epoxy was free from defects thereby preventing leakages into this chamber (Figure 7G).

To demonstrate consistency, we prepared three individual hemoperfusion units (Figure 8A). In the dynamic absorption of endotoxin, it was observed that a significant drop in endotoxin levels was recorded with the first 15 min and tapered off from this point onwards, which was similar to other works [85,86]. As the endotoxin molecules began to occupy the binding sites of PMB, it created a repulsive effect on any free, unbounded endotoxin molecules due to the hydrophobic and ionic forces of the amino groups on the PMB [78,85,87,88]. Comparing the commercially available Toraymyxin and the hemoperfusion system developed in this study, the removal rate and total removal capacity of the PSF MTAM-A-PMB hemoperfusion system was superior (Figure 8B). Clinically, the rapid removal of endotoxin in future patients will significantly arrest the development of sepsis, systemic inflammation [89,90], disseminated intravascular coagulation (DIC) [91], adult respiratory distress syndrome (ARDS) [92], which were often mediated by pro-inflammatory and pro-apoptotic molecules that are released in the presence of endotoxin [14,93]. Finally, the blood component loss post-dynamic absorption, majority of the blood components fell within the acceptable safe limit of 5% [94]. Furthermore, the RBC morphology analysis by the pathology center revealed no abnormality (Figure 8D,E), which suggested that there lumen size of the respective PSF MTAM-A-PMB were sufficiently large to prevent the buildup of sheer force, which will result in hemolysis and coagulation as outlined earlier [95,96].

## 5. Conclusions

The next generation MTAM (PSF MTAM-A) with superior surface area and packing density was successfully developed and fabricated with the above outlined method. The potential of this variant of MTAM remains to be explored for other applications. However, it can only be applied to absorbent based toxin removal applications and not filtration based toxin removal due to the limited surfaces exposed to the outer regions of the membrane.

## Figures and Tables

**Table 1:** The critical solution and fabrication parameters of the respective membrane groups in Figure 3. An interesting trend where the reduction in the surfactant weight percentage to the total shell flow rate ratio appeared to correlate to the changes in the microstructure of the microtube array membranes (MTAMs) from the traditional single layered MTAM to the novel new alternating MTAM.

Membrane Groupc	Polysulfone Concentration (wt%)	Surfactant Concentration (PEG 40, wt%)	Core Flow Rate (mL/h)	Total Shell Flow Rate (mL/h)	Core Flow Rate to Total Shell Flow Rate Ratio	Surfactant to Total Shell Flow Rate Ratio
A	20.0	1.3	7.0	17.0	1.0:2.43	1.0: 13.08
B	20.0	1.3	7.0	15.0	1.0:2.41	1.0:11.50
C	20.0	1.3	7.0	8.0	1.0:1.14	1.0:6.15
D	20.0	2.5	8.0	16.5	1.0:2.06	1.0:6.60
E	20.0	2.5	8.0	14.0	1.0:1.75	1.0:5.60
F	20.0	2.5	8.0	13.0	1.0:1.62	1.0:5.20
G	20.0	7.0	8.0	17.0	1.0:2.22	1.0:2.43
H	20.0	7.0	8.0	15.0	1.0:1.88	1.0:2.14
I	20.0	7.0	8.0	13.5	1.0:1.68	1.0:1.92
J	20.0	8.5	9.0	20.0	1.0:2.22	1.0:2.35
K	20.0	8.0	9.0	18.5	1.0:2.06	1.0:2.32
L	20.0	8.0	9.0	17.0	1.0:1.88	1.0:2.12
